# Enzymatic Processes to Unlock the Lignin Value

**DOI:** 10.3389/fbioe.2018.00020

**Published:** 2018-03-22

**Authors:** Veera Hämäläinen, Toni Grönroos, Anu Suonpää, Matti Wilhem Heikkilä, Bastiaan Romein, Petri Ihalainen, Sara Malandra, Klara R. Birikh

**Affiliations:** MetGen Oy, Kaarina, Finland

**Keywords:** oxidoreductase, laccase, lignin, biomass, lignocellulosic, enzyme

## Abstract

Main hurdles of lignin valorization are its diverse chemical composition, recalcitrance, and poor solubility due to high-molecular weight and branched structure. Controlled fragmentation of lignin could lead to its use in higher value products such as binders, coatings, fillers, etc. Oxidative enzymes (i.e., laccases and peroxidases) have long been proposed as a potentially promising tool in lignin depolymerization. However, their application was limited to ambient pH, where lignin is poorly soluble in water. A Finnish biotechnology company, MetGen Oy, that designs and supplies industrial enzymes, has developed and brought to market several lignin oxidizing enzymes, including an extremely alkaline lignin oxidase MetZyme^®^ LIGNO™, a genetically engineered laccase of bacterial origin. This enzyme can function at pH values as high as 10–11 and at elevated temperatures, addressing lignin at its soluble state. In this article, main characteristics of this enzyme as well as its action on bulk lignin coming from an industrial process are demonstrated. Lignin modification by MetZyme^®^ LIGNO™ was characterized by size exclusion chromatography, UV spectroscopy, and dynamic light scattering for monitoring particle size of solubilized lignin. Under highly alkaline conditions, laccase treatment not only decreased molecular weight of lignin but also increased its solubility in water and altered its dispersion properties. Importantly, organic solvent-free soluble lignin fragmentation allowed for robust industrially relevant membrane separation technologies to be applicable for product fractionation. These enzyme-based solutions open new opportunities for biorefinery lignin valorization thus paving the way for economically viable biorefinery business.

## Introduction

In the biorefinery context, lignin is often mostly regarded as a recalcitrance factor, fermentation inhibitor, sugar stream contaminant, etc. Broader view to the biorefinery, however, considers valorization of lignin a vital component of the economics of the entire concept.

Today, the commercial sales of lignin are limited but growing. Even though the pulp and paper industry produces about 50 million tons of lignin in a year, as defined by Lux Research (Essery, 2017), most of this is burned for power; only 1 million ton reaches the chemicals market. Lignin is currently being used for low- and medium-value applications (e.g., binding and dispersing agents), representing a market of $730 million. As detailed by Lux Research report (Lux, 2014), the total market value for lignin-derived product is at $3.3B, with energy capturing about 89% of the market. Other markets include vanillin production ($192M) and cement additives ($176M). The market has both high-value applications, such as carbon fibers and phenols, as well as lower value applications such as binders and activated carbon. The lowest value use of lignin is its use as solid fuel as energy content of the lignin is in the range of 22 MJ/kg.

The growing need for lignin to produce renewable biochemicals from lignocellulosic feedstocks alone is projected to grow up to 2.9 million Mton in 2017. Potential market value of new lignin-based products is estimated to be about $13.9B by 2020–2025, with lignin-based phenols and carbon fiber poised to capture the largest market potential in the future (Frost and Sullivan, 2014; Smith et al., 2016).

Main hurdles of lignin valorization are its diverse structure and poor solubility. Liquefaction of lignin would allow its use as fuel, as it is reached in high-energy chemical structures. More precise depolymerization or fragmentation of lignin may enable higher value products for various industries from construction to high-performance materials. Notwithstanding the fact that lignin-based replacement products have already been reported (Gandini and Belgacem, 2008; Kim and Kadla, 2010; Laurichesse and Avérous, 2014; Xing et al., 2017) to be useful as binders, coatings, and fillers and others, these applications are not yet widely industrially implemented. Some of the main challenges for full valorization of lignin are the economical production of suitable lignin and maintaining consistent quality throughout different batches.

To achieve desirable properties for the industrial application, lignin usually needs to be fragmented to a lower molecular weight and often chemically modified as well. Some chemo-mechanical methods for lignin depolymerization have been reported (Rinaldi et al., 2016). Different ways to convert lignin to phenolics compounds are liquefaction (Kang et al., 2013), oxidation (Ma et al., 2014), solvolysis (Kleinert and Barth, 2008), hydrocracking (Yoshikawa et al., 2013), and hydrolysis (Roberts et al., 2011). The challenge, however, is that lignin is very difficult to decompose and these processes generate high amounts of solid residue.

Enzymatic degradation of lignin, which occurs in nature, was speculated for a long time to be applicable in the industry (Wong, 2009). This approach seems attractive due to the fact that the catalysis takes place in water and under mild conditions avoiding high pressure, temperatures, and hazardous and expensive chemical catalysts, thus saving CAPEX and lowering environmental impact.

Research efforts for enzymatic lignin depolymerization were especially focusing on laccases (copper oxidases), as these enzymes require no cofactors or cosubstrates (such as hydrogen peroxide), they use oxygen as an electron acceptor and produce water as the only by-product. Prospective and challenges of laccase application in biotechnology were recently reviewed (Roth and Spiess, 2015; Mate and Alcalde, 2017).

Industrially available and most studied laccases are fungal enzymes. These enzymes, however, efficient, mostly work in acidic pH (Rodgers et al., 2010), at which lignin is hardly soluble in water. In addition, fungal enzymes lack the needed thermostability and robustness.

Some studies addressed the feasibility of using these laccases for lignin depolymerization (Munk et al., 2015); however, special measures need to be taken to bring lignin into the soluble state. To this end, laccases were evolved to be used in solvent containing mixtures, ionic liquids, and other technically and economically challenging environments (Mate and Alcalde, 2014; Rehmann et al., 2014). To the best of our knowledge, none of these studies led to an industrially viable enzymatic process for lignin valorization. Alternatively, lignin can be efficiently solubilized in water under alkaline pH. The higher the pH, the higher the lignin solubility. This phenomenon could be exploited in enzymatic depolymerization, where laccases able to function under these conditions.

Bacterial laccases are rather overlooked by the industries due to challenges in their production and lower redox potential as compared to fungal laccases. However, they are also known for larger diversity in operation conditions, including higher thermostability and extended pH range (Santhanam et al., 2011; Rosado et al., 2012). In this respect, they may represent an alternative route to lignin depolymerization—that is by carrying out the process under alkaline conditions.

One of the challenges in laccase application for lignin depolymerization as noted by many experts is the fact that the strong polymerization activity of laccase counters the decomposition of lignin by re-polymerizing the degradation products (Roth and Spiess, 2015). It was postulated (Hahn et al., 2014) that the balance between polymerization and cleavage reactions depend on the structure and redox potential of the laccase, structure and redox potential of the substrate, pH value of the buffer used, incubation temperature, and solvent concentration. Thus, thorough process development is required for directing the reaction into the desired path (Santhanam et al., 2011; Singh et al., 2015). This notion also implies that finding a laccase highly efficient in oxidizing phenolic compounds is not necessarily instrumental for lignin depolymerization. In our experience, highly alkaline pH is more favorable for depolymerization, than acidic conditions.

It has to be noted that even in alkaline conditions lignin may have limited solubility which results in relatively low product titer when compared with chemical processes. Besides, timescale of the enzymatic oxidation poses a challenge, which may be eventually overcome by a continuous process setup with reusable enzyme. Application testing in relevant experimental setup is necessary to evaluate the industrial relevance.

In this study, we present a genetically engineered commercially produced bacterial laccase which is functional and stable at pH as high as 10–11 and demonstrate its use in reactor-scale lignin depolymerization.

## Materials and Methods

### Enzyme Activity Testing with Standard Substrates

Relative laccase activity at different pH with standards substrates, 2,2′-Azino-bis(3-ethylbenzthiazoline-6-sulfonic acid) (ABTS), syringaldazine (SGZ), and 2,6-dimethylphenol (DMP), were determined as follows. Substrates were prepared as 10× stock solutions: 50 mM ABTS and 10 mM DMP in water and 250 µM SGZ in methanol. Enzyme was diluted to provide linear product accumulation during the reaction time. Reaction mixtures were prepared by adding 10 µl of enzyme to 170 µl of 30 mM Britton & Robinson buffer (pH 2–11) and equilibrated to room temperature in a 96-well microtiter plate. The enzymatic reactions were initiated by adding 20 µl of substrates solutions and oxidation of substrates was followed by monitoring absorbance at 405 nm for ABTS, 450 nm for DMP, and 530 nm SGZ. Relative reaction rates were calculated from the absorbance increase vs time.

Specific enzyme activity was determined using ABTS as substrate. Reaction mixture was prepared by mixing 20 μl of diluted enzyme with 430 μl of 100 mM sodium acetate pH 4.5 in 1-cm light-path spectrophotometer cuvette, the mixture was equilibrated to 60 C; the reaction was initiated by adding 50 μl of ABTS solution and incubated at 60 C; UV measurements at 405 nM were performed every 2 min. One unit of laccase activity was defined as the enzyme amount oxidizing 1 μmol of ABTS per minute under these conditions (whereas extinction coefficient of ABTS at 405 nm is 36,800 M^−1^ cm^−1^). One microkatal is the amount of enzyme oxidizing 1 μmol of substrate per second, hence 1 μkat equals to 60 U.

### Laccase Stability Testing

Laccase stability at different pH was determined by pre-incubation of the enzyme at a certain temperature and pH, followed by measurement of residual activity.

For this, 20 µl of enzyme was added to 180 µl of 150 mM Britton & Robinson buffers with the desired pH (9–11) pre-equilibrated to the desired temperature, incubation was continued for the desired time. Samples of 10 μl were taken at certain time points (starting from time 0) and immediately added to 170 µl of 100 mM sodium acetate buffer pH 4.5 in a 96-well microtiter plate and kept on ice. When all the time-point samples were collected, enzymatic reactions in the microtiter plate were started by adding 20 µl of 50 mM ABTS solution, and relative reaction rates were determined by monitoring absorbance at 405 nm vs time.

### Alkali Soluble Lignin Preparation

Hardwood lignin extracted by extrusion-based process was obtained from an industrial source (http://sweetwater.us/process/). As reported by the manufacturer, the molecular weight is Mn = 2,700–4,000 g/mol, *M*_w_ = 7,500–8,280 g/mol, PDI = 2.2–3.0, and the hydroxyl content is 2.85 mmol/g aliphatic -OH (possibly carbohydrates), 0.9 mmol/g syringyl -OH, 0.53 mmol/g guaiacyl OH, 0.1 mmol/g phenolic OH.

Alkali soluble lignin was prepared as follows: lignin was solubilized at 100 g/l in 0.25 M NaOH, mixed for 30 min at room temperature, centrifuged at 6,000 *g* for 20 min, supernatant was dried in oven at 105 C, and stored at room temperature until used. Drying of lignin after alkaline solubilization was not essential for laccase oxidation, it was performed for convenience in storage and obtaining reproducible results with different batches of lignin. For the laccase treatment, dried alkaline soluble lignin was dissolved at 25 g/l in water (solubilization was complete) and pH adjusted to 10.5.

### Lignin Depolymerization with Laccase MetZyme^®^ LIGNO™

Alkaline soluble lignin prepared as described earlier was dissolved in water to the final concentration 25 g/l. The reactions were run in 1 l twin-pot bioreactor (BIOSTAT^®^ B plus twin from Sartorius Stedim Biotech). Mediator syringaldehyde was added 0.1% w/w in respect to lignin and the reactor equilibrated to 50°C, and pH 10.5 with aeration 0.16 l/min. After that, the enzyme was added 80 nkat/g of lignin (~5 U/g of lignin). Control reaction was run without the enzyme—further throughout the text referred to as “Control lignin” or “Control sample.” Reaction was continued for 21 h with constant aeration (0.16 l/min, dissolved oxygen level was remaining constant from the time of enzyme addition), pH was controlled with NaOH to remain 10.5 throughout the reaction.

### Size Exclusion Chromatography

Size exclusion chromatography for lignin samples and molecular weight standards was performed using HPLC chromatographer 120 CompactLC with UV detector (Agilent Technologies), equipped with size exclusion column MCX 1,000 Å 5 µm, 8 mm × 300 mm and with pre-column MCX 5 µm, 8 mm × 50 mm (Polymer Standards Service). Isocratic mode with 0.1 M NaOH eluent flow 0.5 ml/min at RT was used; run time was 40 min. Lignin samples were monitored at 280 nm. Molecular mass standards (polystyrene sulfonate sodium salt standards Mp = ~0.9 to ~65 kDa, Polymer Standards Service) were monitored at 254 nm. Data were acquired using the EzChrom Elite Compact software.

Primary HPLC traces acquired using the Agilent EZChrom Elite software were transferred to tailor-made MS Excel(R) spreadsheets for further processing. Signal vs retention time graphs were produced from 1 Hz time series to depict the chromatography traces.

Polystyrene sulfonate molecular mass standards (Polymer Standards Service), ranging in mass at peak maximum from *M*_W_ = ~900 to ~65,000, and syringaldehyde (*M*_W_ 182) were used for calibration of molecular weights.

Based on HPLC profiles, average molecular weights—number average molecular weight (*M*_n_) and the weight average molecular weight (*M*_w_), which emphasizes the contribution of polymers with larger molecular weights, were calculated for lignin fractions.

The *M*_n_ was calculated by dividing the total relative polymer weight by the total number of polymer molecules, using the following equation:

$$M_{\text{n}}=\frac{W}{\sum N_{i}}=\frac{\sum \left(M_{i}N_{i}\right)}{\sum N_{i}}=\frac{\sum \left(H_{i}\right)}{\sum \left(H_{i}/N_{i}\right)}.$$

The *M*_w_ was calculated using the equation:

$$M_{\text{w}}=\frac{\sum \left(W_{i}M_{i}\right)}{W}=\frac{\sum \left(H_{i}M_{i}\right)}{\sum H_{i}}$$

where *W*—total weight of polymers, *W_i_*—weight of i*-*th polymer, *M_i_*—molecular weight of the *i-*th elution time, *N_i_*—number of molecules with molecular weight *M_i_, H_i_*—height of *i-*th elution time.

### Demethylation Detection

Demethylation of lignin was followed by monitoring methanol in the reaction mixture after completion of the oxidation. Purpald method adapted from Anthon and Barrett (2004) was used for detecting methanol. The method is based on conversion of methanol to formaldehyde by alcohol oxidase enzyme and subsequent spectrophotometric quantification of the formaldehyde. Alcohol oxidase from *Pichia pastoris* and Purpald reagent (4-amino-3-hydrazino-5-mercapto-1,2,4-triazole) were purchased from Sigma Aldrich.

Purpald method was adapted to 96-microwell plate (Spectra Plate, Cat. 6005640, Perkin Elmer). Lignin samples were adjusted to pH 7.0 with HCl and diluted 1:25 with water. Methanol standards (0–0.5 mM) were used. For all lignin samples, negative controls were prepared by adding buffer without alcohol oxidase. Diluted lignin samples (28 µl) or methanol standards were pipetted as triplicates into 96-well plate, and 28 µl of 100 mM sodium phosphate buffer pH 7 containing 1 U/ml alcohol oxidase (solution prepared just before use) was added to the wells. The mixtures were incubated at 30°C for 10 min with 250 rpm shaking (MaxQ 4450, Thermo Scientific). Purpald reagent (34 mM in 0.5 M NaOH) was prepared just before use, and 56 µl of Purpald reagent was added to the reactions; the samples were further incubated at 30°C for 30 min with 250 rpm shaking. After that, 168 µl of water was added to the wells, mixed for a minute, and absorbance measured at 560 nm using spectrophotometer (Multiscan Go, Thermo Scientific). The methanol concentrations in the lignin samples were calculated using absorbance values obtained from the calibration curve of methanol standard solutions.

### Lignin Solubility Assessment

Lignin sample after enzyme treatment (or control sample, run without the enzyme) was equilibrated to room temperature and showed initial pH 10.8 (as depolymerization was carried out at 50°C at pH 10.5). Sample was slowly titrated with HCl to allow the pH to stabilize at integer values (10.0, 9.0, 8.0, and so on); aliquots were taken at each of these pH and frozen. After completing the titration, all samples were thawed at room temperature and allowed to stand for 1 h, after which samples were centrifuged at 18,000 *g* for 30 min and UV absorption was measured in the supernatant to determined soluble fraction. Relative values to the starting material were plotted against the pH.

### Lignin Fractions Separation

For lignin fractions separation, ultrafiltration was performed using tangential flow membrane units Vivaflow 200 (Sartorius) with polyethersulfone membranes with 10- and 2-kDa cutoff values. The units were used according to the manufacturer’s protocol (2.5 bar pressure, 200–400 ml/min retentate flow/module). The ultrafiltration was carried on until penetrate volume reached 90% of the starting material volume.

### Lignin Particles Measurement

Dynamic light scattering (DLS) measurements were performed at 298 K using a zetasizer equipment (Model Nano ZS, Malvern, Worcestershire, UK) equipped with a red laser operating at 632.8 nm (He/Ne laser) and the detector positioned at 173° (non-invasive back scattering technology). The measuring size range of 0.1 nm to 10 µm was applied. The data were analyzed using the Malvern Zetasizer Software v. 7.12. Soluble lignin samples were prepared and treated with the enzyme as described above and diluted 1:100 with 150 mM Britton and Robinson buffer pH 10.5 for the measurement.

## Results

### Alkaline Laccase MetZyme^®^ LIGNO™

**Figure 1:**
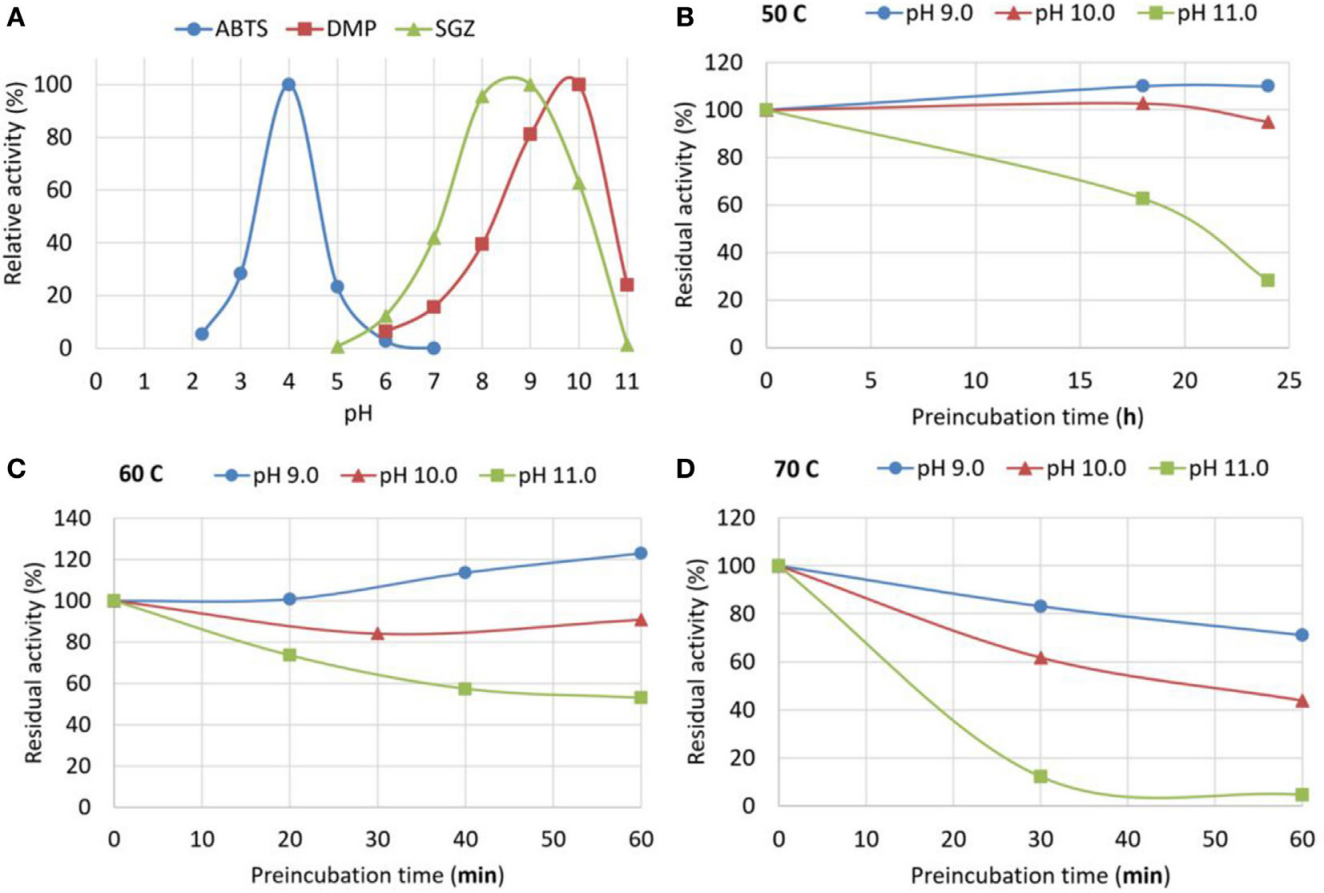
Standard activity and stability of MetZyme^®^ LIGNO™. **(A)** pH dependence of MetZyme^®^ LIGNO™ activity toward standard low-molecular weight substrates: 2,2′-Azino-bis(3-ethylbenzthiazoline-6-sulfonic acid) (ABTS), syringaldazine (SGZ), and 2,6-dimethylphenol (DMP). **(B–D)** Stability of MetZyme^®^ LIGNO™ at various pH (9–11) and temperatures (50, 60, and 70°C); pre-incubation time at indicated conditions is plotted against residual activity measured after pre-incubation. Pre-incubation at 50°C was carried out over a span of 24 h, at 60 and 70°C—over 1 h; temperatures and pH are indicated over the graphs. The curves do not represent a model fit, they are line interpolations that Microsoft excel provides as a default and they are added only for convenience.

New commercially available genetically engineered bacterial laccase MetZyme^®^ LIGNO™ (MetGen) was tested with following standard laccase substrates: 2,2′-Azino-bis(3-ethylbenzthiazoline-6-sulfonic acid) (ABTS), SGZ, and DMP. Laccase efficiency in oxidizing different substrates depends on both—laccase properties and substrate properties (Rosado et al., 2012), thus optimal pH needs to be determined for a particular laccase with each substrate. As seen from Figure 1A, the enzyme is able to oxidase phenolic substrate (DMP) at extremely alkaline pH, as high as 11.

Enzyme stability may be severely compromised in alkaline pH, and standard activity measurements can be misleading, as they are performed in short-time reactions. Enzyme stability at alkaline pH was evaluated by pre-incubation of the enzyme in a buffer with desired pH and further testing the residual activity after readjusting the pH (Figures 1B–D). ABTS was used for residual activity measurement as it provides the highest sensitivity and dynamic range. The enzyme was found to be fairly stable in the tested pH range at 50°C, therefore pre-incubation at this temperature was carried out over an extended period of time—24 h (Figure 1B); in other temperatures 1 h pre-incubation was used.

### Treatment of Alkali Soluble Industrial Lignin with MetZyme^®^ LIGNO™

**Figure 2:**
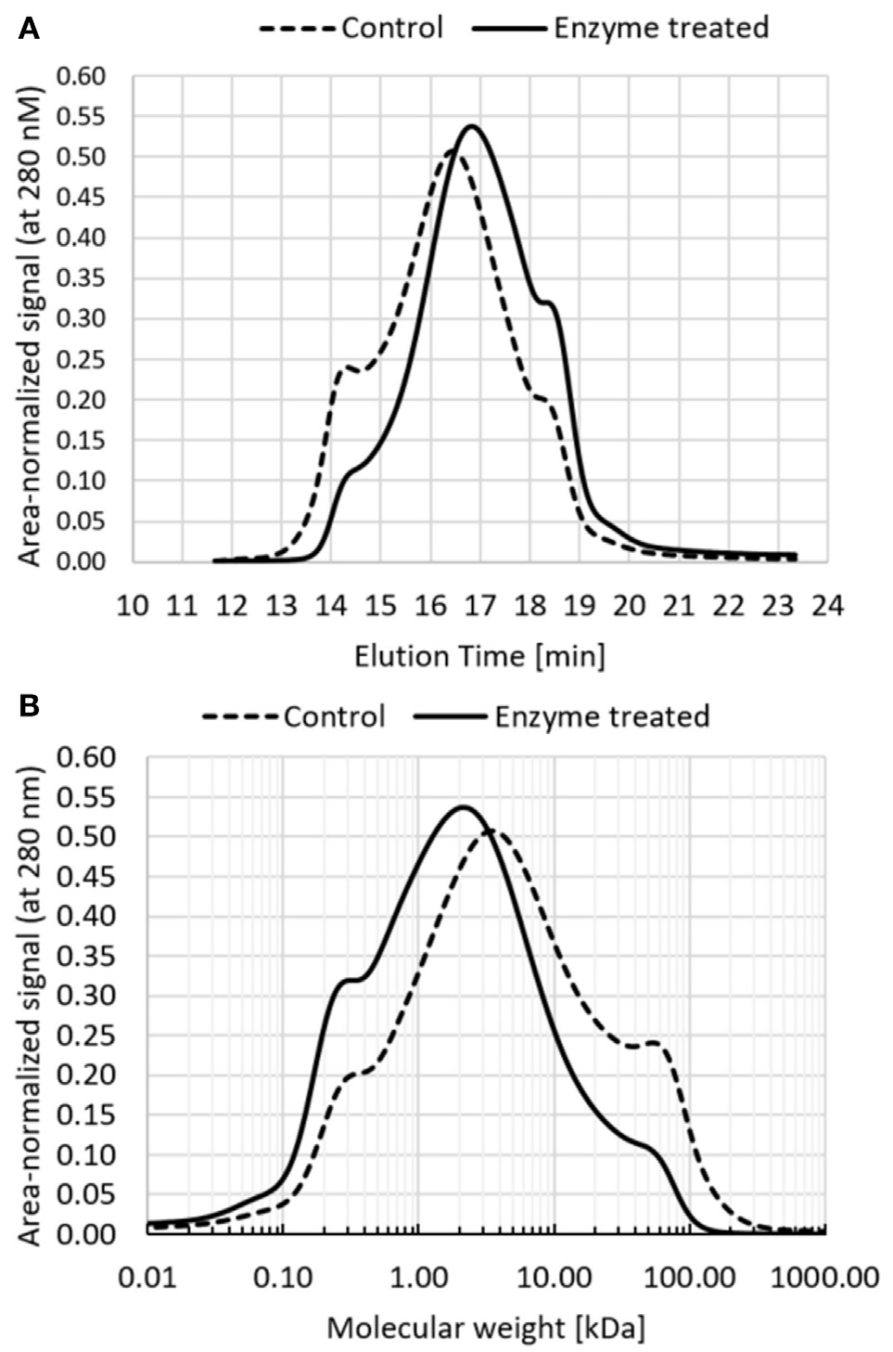
Molecular weight distribution of enzyme-treated lignin sample and control sample (the same process run without the enzyme). **(A)** Elution profiles of size exclusion chromatography, profiles were area-normalized to reflect the same total mass; **(B)** molecular weight distribution calculated based on HPLC profile using molecular weight standards.

Industrial biorefinery lignin obtained from extrusion pretreatment method was used in these experiments. Experiments were performed in a reactor scale and final samples were analyzed by size exclusion chromatography with UV detector set to 280 nm (Figure 2A). Based on the HPLC profile, molecular weight distribution was obtained using molecular weight standards (Figure 2B). The results show a substantial transformation of the molecular weight distribution toward lower molecular weights.

### Properties of Enzyme-Treated Lignin

**Figure 3:**
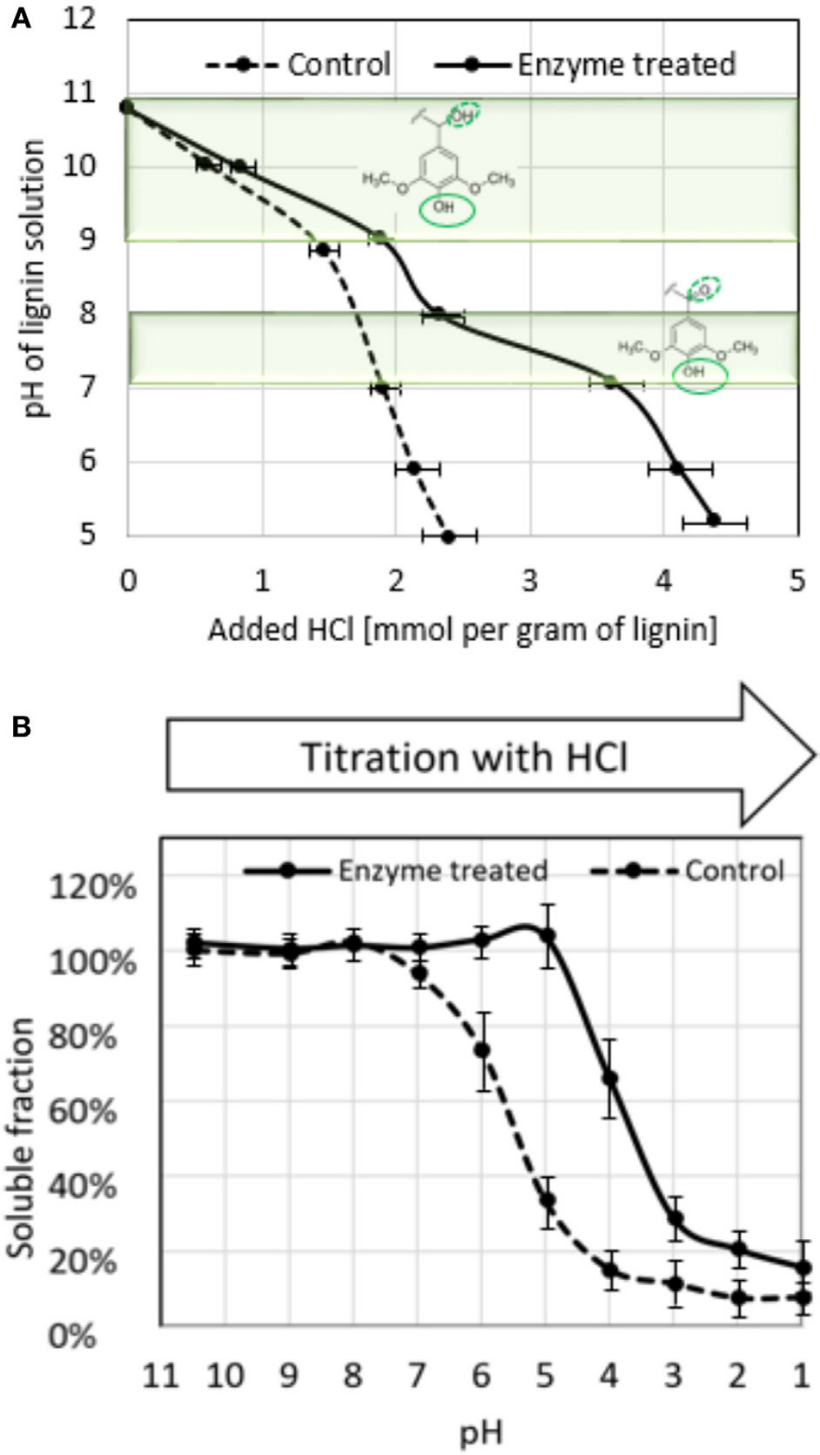
Properties of enzyme-treated lignin revealed from acid titration. **(A)** Acid titration curve of enzyme-treated and control lignin; highlighted areas indicate pH ranges of deprotonation of phenolic hydroxyls (pH 9–11) and phenolic hydroxyls of lignin units with keto group at alpha-carbon (see Discussion), deprotonating hydroxyls in the molecules are outlined with solid line, substituent at alpha-carbon is outlined with dotted line; **(B)** solubility of enzyme-treated and control lignin at different pH. The results are average of three independent titrations.

Enzyme-treated lignin and control sample were subjected to titration with hydrochloric acid and the acid consumption was recorded throughout the course of the titration (Figure 3A). Enzyme-treated sample showed more extensive buffering in the range of pH 9–11, corresponding to protonation of phenolic hydroxyls, and an additional buffering range around pH 8–9, likely to reflect the presence of C-alpha oxidized phenolic groups as pictured on the graph of Figure 3A (see Discussion).

During the titration, it was observed that enzyme-treated lignin starts to precipitate at much more acidic pH than control sample. To evaluate this phenomenon, we prepared lignin samples by adjusting the pH of control and enzyme-treated preparations (25 g/l) to different pH values, and monitored lignin precipitation (Figure 3B). Enzyme-treated lignin remained fully soluble even at pH 5.0, whereas in control sample at this pH only 35% of lignin remained in solution.

Oxidative demethylation of lignin is cleavage of aryl ether between phenolic oxygens and methoxy carbons, resulting in formation of a methanol molecule and a new phenolic hydroxyl group. It is a desirable process in lignin upgrade, as it increases the number of hydroxyls and thus results in activation of lignin. Demethylation was monitored by measuring MeOH in the reaction mixture after depolymerization. The enzyme-treated sample showed 0.47 mmol of MeOH per gram of lignin as opposed to 0.11 mmol/g in the control sample. This corresponds to a 30% increase of total number of phenolic hydroxyls when compared with the starting material as of manufacturer specification (1.5 mmol/g). It has to be noted that this method may result in some underestimation of the degree of demethylation, as MeOH is likely to be evaporated during the course of lignin depolymerization in spite of the water condensation unit present in the reactor. Nevertheless, this result confirms an important notion that significant demethylation does take place during this process.

### Dynamic Light Scattering

As it has been shown previously, lignin tends to be dispersed in aqueous medium and the particle size depends on its chemical composition, solubility, pH, and other factors (Nistor et al., 2014). This property is important for evaluating lignin as potential dispersant for industrial applications.

**Figure 4:**
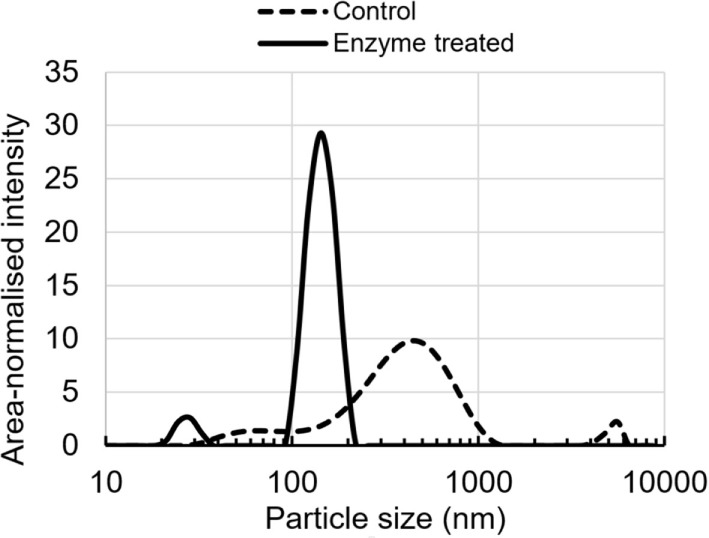
Colloidal particle size distribution of enzyme-treated and control lignin in a buffer at pH 10.5.

The particle size distribution curves show a clear differentiation between untreated and enzymatically treated lignin (Figure 4). Particle size distribution curve is multimodal in the case of unmodified lignin sample, showing relatively broad main peak at 459 nm and two other peaks at 68 and 5,560 nm. On the other hand, the enzymatically treated sample shows bimodal distribution with main peak at 142 nm and lower intensity peak at 28 nm. In addition, the peaks are clearly narrower than those for untreated lignin, suggesting less polydispersity.

The observed differences in the particle size distribution and polydispersity between untreated and enzymatically treated lignin samples indicate an increased solubility and better dispersibility for the latter.

### Fractionation of Enzyme-Treated Lignin

**Figure 5:**
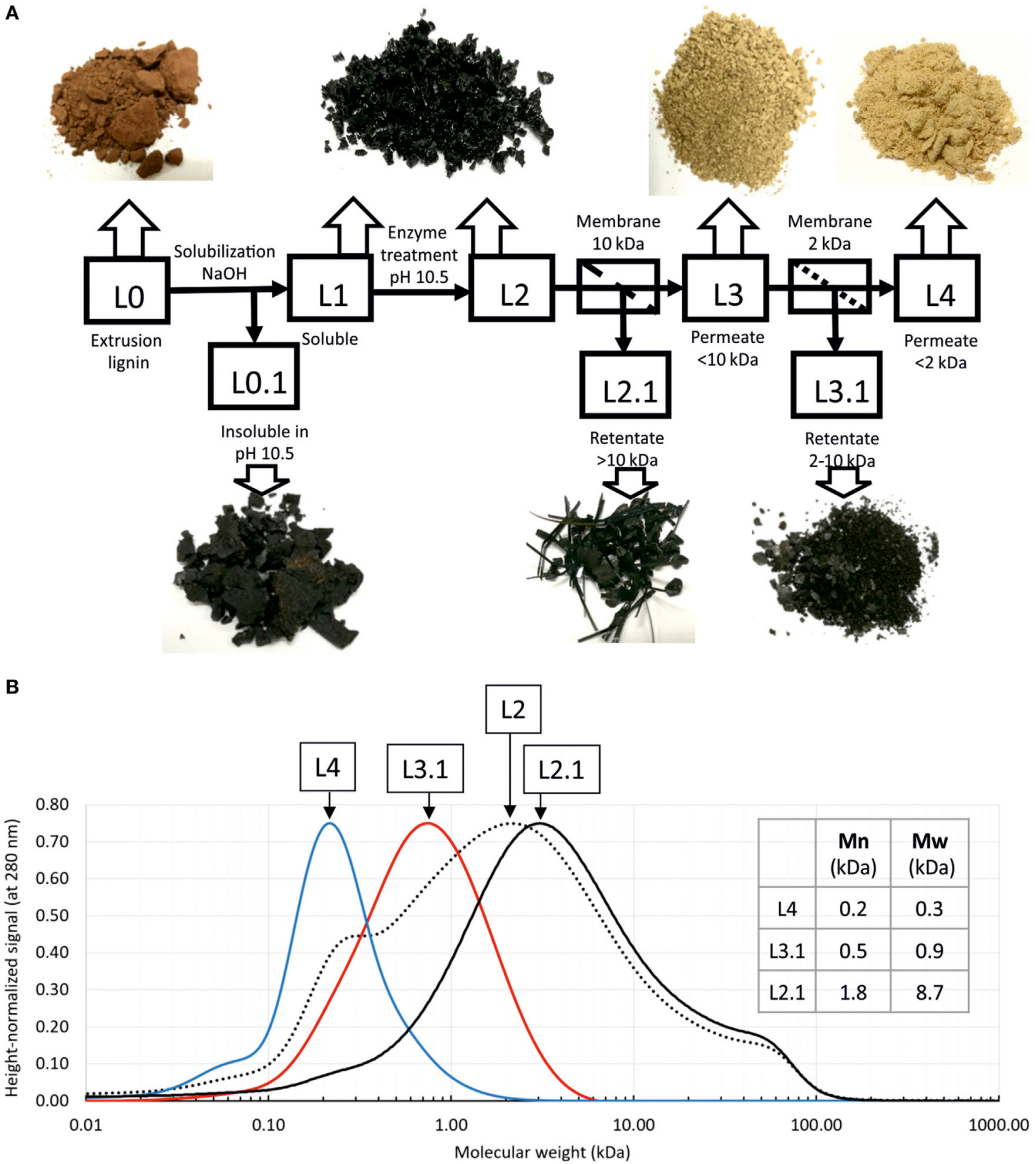
Fractionation of enzyme-treated lignin. **(A)** Experiment flow chart and lignin fractions (dried); **(B)** analysis of lignin fractions by size exclusion chromatography. Calculated average molecular weights are given in the table. Since claimed column resolution range is 100–100,000 Da, and molecular weight standards range starts from 600 Da, smaller molecular weights may not be calculated correctly.

Enzyme-treated lignin was further subjected to ultrafiltration to separate fractions of different molecular weight. The experiment flow chart is depicted in Figure 5A. Fractions were designated with codes (L0 for the extrusion lignin, L1—for alkaline soluble fraction, and so on according to the Figure 5). Further mentioning of these codes refers to the corresponding lignin fractions.

As seen on the photographs (Figure 5A), dried lignin fractions have different color and morphology. Lignin fractions were analyzed by size exclusion chromatography and showed good separation and low dispersity (Figure 5B). Average molecular weights *M*_n_, and *M*_w_ were calculated for selected fractions. Notably, from this analysis L4 fraction contains predominantly molecules no bigger than trimers. More precise determination of molecular weights of this smallest fraction requires different type of column.

## Discussion

In the view of rising BioEconomy, proper exploitation of lignin through more noble applications than fuel are of vital importance.

The key characteristics of lignin that need to be achieved are unification of its molecular weight and increased reactivity. In addition, the material needs to be transformed to a more manageable form—soluble or melting. These characteristics largely depend on the lignin origin and preparation methods and can be further tuned after lignin separation (Abdelaziz et al., 2016; Rinaldi et al., 2016); this is often referred to as lignin upgrade. Methods for isolation and chemical oxidative upgrade of lignin were recently reviewed by Lange et al. (2013). It is evident that different strategies of lignin valorization have been exploited from trying to preserve its native structure and high-molecular weight to depolymerizing it to monomers (Gouveia et al., 2012; Picart et al., 2017).

It is important to understand that lignin preparations of different molecular weight can be further valorized and utilized in various industrial applications (Gandini and Belgacem, 2008; Laurichesse and Avérous, 2014) as long as chemical/physical properties are matching the requirements (Passoni et al., 2016). Thus, the target of lignin depolymerization is to create lignin fractions that are bioequivalent, for example, to oil-based compounds used as resins, adhesives, composites, and foams (Table 1). Generally, if higher value chemicals are wanted to be replaced, stricter molecular weight distributions are required—reachable *via* enzymatic lignin depolymerization. In this view, it is not necessarily desirable to depolymerize lignin to monomers in its entirety. We believe that it is beneficial from the economic and environmental point of view to pursue a less energy intensive transformation of the starting material, followed by efficient separation, which will provide valuable fractions for various applications. In this view, enzymatic approach seems very attractive.

**Table 1:** Valorization of lignin by oxidative upgrade and fractionation.

	Molecular weight	Reactivity and solubility	Application	Bioequivalent of	Approximate of the oil-based chemical
Pulp and paper lignin	5–100 kDa	Poor	Burning	Oil/electricity	50–150 €/ton
Dissolved lignin/biorefinery lignin	3–50 kDa	Medium	Resins and adhesives	Formaldehyde	400–500 €/ton
Enzyme oxidized lignin	Custom	Good	Composites	Polymers	>1,000 €/ton
Enzyme depolymerized lignin	0.3–2 kDa	Good	Foams and composites	PVA	1,000–1,500 €/ton
Enzyme depolymerized fractions	0.3, 0.5, 0.7, …, 2 kDa	Very good	New materials	Specialty chemicals and polymers	>1,500 €/ton

In this study, for the first time, enzymatic treatment of bulk industrial lignin was demonstrated at extremely alkaline conditions, where lignin is soluble in water.

This has become possible due to a new industrial genetically evolved laccase of bacterial origin with exceptional properties. As demonstrated in Section “Results,” the enzyme retains full activity after 24 h incubation at 50°C at pH 10 and 30% activity at pH 11.

In a one-liter bioreactor scale, extensive lignin depolymerization was observed, accompanied by chemical activation *via* demethylation and benzylic oxidation as well as increased solubility in neutral and acidic pH and altered colloidal behavior. In the view of recent advancements in using lignin nanoparticles for grafting synthetic polymers and manufacturing thermoplastic composites, the particle size decrease and unification that we observed in enzyme-treated samples may be advantageous for this technology (Hilburg et al., 2014).

In the course of process optimization, we observed that the balance between polymerization and depolymerization reactions is pH and aeration dependent. Under highly alkaline conditions and strong aeration, depolymerization prevailed over polymerization (data not shown).

We were further able to separate lignin fractions with defined molecular weight and low dispersity. Dried lignin fractions have district appearances. Notably, lignin fraction of >10-kDa molecular weight (L2.1 in Figure 5) shows a clear directional drying pattern and appears as stripes and elongated flakes, as opposed to the full sample (L2 in Figure 5), which dries as small rounded grains. Our results also show that 2-kDa cutoff membrane allows for separation of predominantly monomeric lignin.

Importantly, the absence of organic solvents in the reaction mixture allowed for utilization of polymer-based ultrafiltration membranes, which are widely used in food industry, making scalable and economically feasible. Ultrafiltration membranes of different cutoff are available and widely used in industry. The choice of membranes used in this study was rather arbitrary and can be tuned to the targeted lignin size.

The mechanism of lignin oxidation by bacterial laccases was thoroughly described by Rosado et al. (2012). As opposed to fungal laccases that oxidize the phenolic substrates in the phenolic form, bacterial laccases oxidize them optimally in the phenolate form, i.e., after deprotonation of the phenolic group. Structurally, this is due to the fact that bacterial laccases (such as COTA) do not contain any negatively charged residue in the vicinity of the substrate binding site, and the efficiency of the oxidation of phenols rely mostly on the protonation/deprotonation state equilibria of the compounds themselves. Thus, oxidation is strictly dependent on the chemical nature of the substrates themselves, i.e., on their pKa values.

The pKa values of phenolic mediators such as syringaldehyde, acetosyringone, and methyl syringate lie between 7 and 9. This outlines the range where bacterial laccases could be effective in oxidizing lignin *via* a mediator. However, pKa of phenolic groups in lignin itself is in the range of 10–11. This makes lignin poorly soluble at pH values substantially lower than this range and also prevents or largely impedes direct oxidation of lignin phenolics by a bacterial laccase. Therefore, reaction conditions exploited in this study open a whole new mode of operation for enzymatic lignin oxidation.

Another important aspect of laccase-mediated oxidation of lignin is that not only it considerably changes molecular weight of the molecules but also changes its properties. Significant increase in water solubility of enzyme-treated lignin was observed even at neutral and acidic pH as illustrated in Figure 3B. Paralleled to this, enzyme-treated lignin demonstrated altered behavior of in acid titration. It showed a specific span of buffering capacity at pH 7–8, that was absent in the control sample, indicating the appearance of new moieties with pKa in this range.

**Figure 6:**
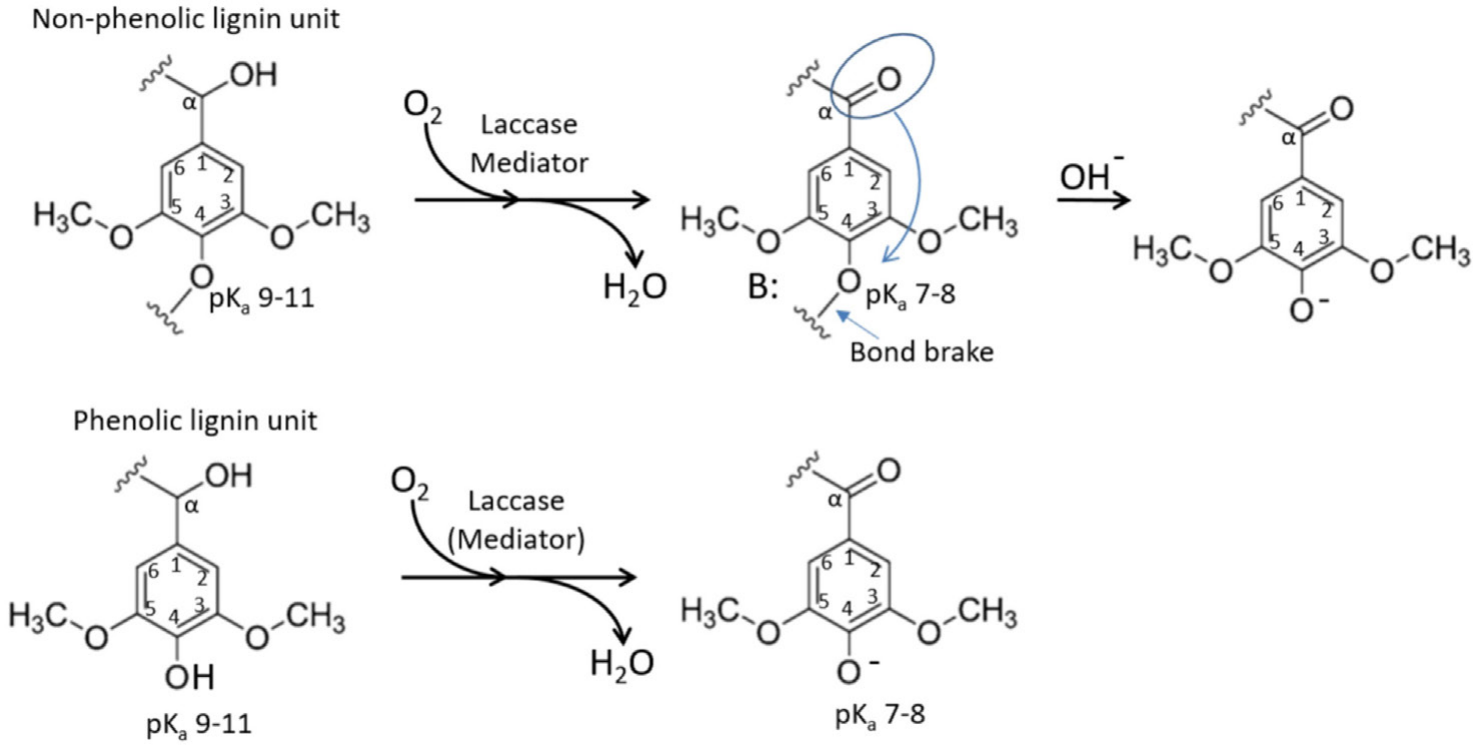
Schematic representation of laccase driven oxidation of a lignin unit. Oxidation of alpha-carbon to a ketone lowers pKa of the corresponding C1 hydroxyl, resulting in increased solubility in neutral-to-acidic pH and facilitated lignin chain brake.

To interpret this observation, one has to revert to the reaction mechanism of enzymatic lignin oxidation. According to various scenarios following the radical transfer from the mediator to a lignin unit (phenolic or non-phenolic), reported in previous works, one of the major outcomes of this process is benzylic oxidation resulting in a ketone (or aldehyde) groups at the alpha-carbon of the lignin unit (Figure 6). Further chemical scenarios elucidated from the experiments with lignin model compounds mainly revolve around the spacer between benzylic units where the oxidized alpha-carbon is residing (Lahtinen et al., 2009; Ohashi et al., 2011) and little attention is paid to the para-position of the phenyl group connected to this alpha-carbon (C4 position of the same benzene ring). Rightfully so, as in acidic pH, where conventional laccases are operating, that position is not yielding any pathways of interest. However, in alkaline pH, this position gains importance.

Ragnar et al. (2000) reported pKa values for phenolic C4 hydroxyls for over 50 different lignin model compounds including monomers and dimers with various substituents at C1. This remarkable data collection clearly shows that pKa of the C4 phenolic hydroxyl remains in very alkaline region (between 9.5 and 11) practically in any environment of the corresponding C1 atom, apart from alpha-carbon being in the form of a ketone or aldehyde (which is the case upon laccase oxidation). In the later situation, pKa of C4 hydroxyl drops to a value between 7 and 8. This could explain the buffering of oxidized lignin at this pH range as well as increased solubility at neutral-to-acidic pH. This is because in oxidized phenolic units, C4 hydroxyl would tend to remain deprotonated at lower pH (Figure 6). Another implication of this shift in pKa is that the corresponding alpha-carbon oxidized non-phenolic unit with an ether bond at C4 carbon (Figure 6 top) represents a good leaving group (Boyd, 1985) and may promote lignin chain brake (Figure 6 top, right) or demethylation event.

At the same time, increased number of other types of phenolic units in the oxidized lignin is reflected by the expanded buffering capacity at pH 9–11 (Figure 3A). In the enzyme-treated sample, the acid consumption in this pH range was increased by 0.5 mmol/g of lignin, which coincides with the degree of demethylation determined by methanol detection. In this respect, titration curve of the control sample is in agreement with lignin specification obtained from the manufacturer (1.5 mmol/g of total aromatic hydroxyls), confirming our interpretation.

This study is not focusing on polymerization of lignin using laccase; however, we did observe re-polymerization of the shorter molecules when pH is lowered, especially under limiting aeration. Under these conditions, the reaction was apparently driven more toward polymerization. This also represents an industrial opportunity, as chemically activated high-molecular weight lignin is highly desirable for some high-end bio-based materials. In addition, polymerization may provide a tool for removing small lignins from the process by polymerization followed by sedimentation or filtration. This is certainly an important aspect for future investigation.

Further studies need to be conducted to fully characterize the enzyme-treated lignin in terms of chemical composition and reactive groups as well as its applicability in industrial processes.

## Author Contributions

KB, VH, and AS contributed to enzyme discovery engineering and characterization. TG and MH contributed to lignin treatment, characterization, and separation. SM contributed in size exclusion chromatography analysis. PI contributed to colloidal particles analysis. BR implemented computational analysis of HPLC data and contributed to enzyme production. KB wrote the manuscript with support of all the coauthors and offered the interpretation of the results. All authors discussed the results and contributed to the final manuscript.

## Conflict of Interest Statement

VH, TG, AS, MH, BR, PI, and KB were employed by the company MetGen Oy. All other authors declare no competing interests.
